# Coexistence of Cervical Disc Herniation and Shoulder Soft Tissue Pathologies and the Effect of Sleeping Positions and Orthopedic Pillows

**DOI:** 10.7759/cureus.44510

**Published:** 2023-09-01

**Authors:** Hidayet Safak Cine

**Affiliations:** 1 Neurosurgery, Istanbul Medeniyet University, Prof. Dr. Suleyman Yalcin City Hospital, Istanbul, TUR

**Keywords:** sleeping positions, shoulder pain, neck pain, orthopedic pillow, cervical disc herniation

## Abstract

Background/aim

This study investigates the degree of coexistence of cervical disc herniation and shoulder soft tissue pathology, as well as the effect of sleeping positions and orthopedic pillow use.

Materials and methods

This present study was conducted on 72 patients with shoulder/arm pain operated on for cervical disc herniation. Two groups were examined according to the presence of shoulder soft tissue pathology, four common sleeping positions, and the use of an orthopedic pillow. Preoperative and postoperative shoulder/arm visual analog scale (VAS) scores were compared.

Results

The preoperative VAS values were 7.35, while the postoperative VAS values were 3.32. Twenty-one patients (29.2%) had a disc at the C3-4 level, a rate equal to that for the C5-6 level. Twenty-four patients (33.3%) had a disc at the C4-5 level. Thirty-two cases (44.4%) slept in a side-lying position on the same side as their disc herniation. Among those with a herniated disc at the C3-4 level, 8 (53.3%) preferred sleeping side-lying on the opposite side of the disc. In contrast, those with a herniated disc at the C4-5 level more frequently (40.6%) slept side-lying on the same side as the disc. Mean VAS scores were significantly higher in cases with shoulder soft tissue pathology and were significantly lower in the group that used orthopedic pillows (p<0.001).

Conclusion

Shoulder soft tissue pathologies should be considered in postoperative shoulder pain. The use of orthopedic pillows is effective in preoperative and postoperative pain. Sleeping positions do not affect the shoulder/arm pain before and after the operation, but they affect the level of cervical disc herniations.

## Introduction

Neck pain is the fourth leading reason of disability, with an annual prevalence of over 30%. Acute neck pain disappears with or without treatment most of the time. However, almost 50% of individuals continue to experience some degree of pain or frequent pain [1]. Neck pain is a multifactorial but treatable disease. Several population-based studies have revealed the role of various modifiable and non-modifiable risk factors for neck pain, such as advanced age and being female [2], as well as lack of physical activity and duration of daily computer use [3]. Humans spend about a third of their life during their sleeping period. Sleep quality and neck pain can be reciprocal as both can lead to the other [3]. Some studies have shown the importance of physical exercise. Regular stretching can reduce neck and shoulder pain and improve quality of life with neck or shoulder pain [4]. Cervical spondylosis, fibromyalgia, and cervical radiculopathy are common disorders in the cervical region that can cause neck pain [5]. Cervical spine disc herniation is a disabling source of cervical radiculopathy [6]. The annual incidence of cervical disc herniations accounts for 18 per 100,000 residents. The operation is needed for 26% of the cases within five years during the follow-up [7]. 

Shoulder pain has diverse reasons, such as local soft tissue, articular, or bone pathologies. Shoulder pain may be referred from the neck or shoulder that may be difficult to distinguish clinically from those localized to the shoulder [8]. It can be termed as dual pathology when these two pathologies are seen at the same time. This dual pathology can be the reason for residue postoperative shoulder/arm pain after cervical disc herniation. 

The types of pillows, specifically spring and rubber, have been proven effective in reducing neck pain and increasing pillow contentment in patients with chronic neck pain [9]. Although sleeping in a side-lying position can cause a decrease in blood supply to the shoulder, resulting in shoulder pain [10], there may be no change in the alignment of the cervical spine in a side-lying position regardless of pillow type [11]. This present study evaluated the coexistence of cervical disc herniation and shoulder soft tissue pathology in relation to sleep positions and their effect on postoperative pain. Correlatively, it aimed to determine the impact of orthopedic pillows on these pathologies.

## Materials and methods

Study populations

Our study conforms to the ethical rules of the World Medical Association Helsinki Declaration and is approved by the Ethics Committee decision for clinical research. This study comprises all the patients with a single-level cervical disc herniation admitted to the Neurosurgery Clinic between June 2018 and September 2022. The data of the patients have been collected retrospectively. Patients with a single-level cervical disc herniation operated on for this cause were included in this study. Only those presented with shoulder/arm pain on one side were included in the study. Cervical disc herniations that cause bilateral pain were excluded from the study. The other chronic or rheumatological reasons, genetic spinal deformities that can cause shoulder/arm pain, were excluded. Patients not operated on for cervical disc herniation were excluded from the study. Patients with spinal deformities affecting sleeping position or patients who could not change position due to limb paralysis were not included in the study. 

All patients underwent a single-level anterior cervical discectomy and fusion (ACDF) operation. The criteria for surgery included arm/shoulder pain that did not resolve despite medical treatment and extruded cervical disc herniation detected by cervical magnetic resonance imagining (MRI). All patients received non-steroidal anti-inflammatory drugs (NSAIDs) and muscle relaxants for medical treatment. As a standard conservative treatment, 400 mg of phenyramidol and diclofenac sodium 100 mg of oral therapy were given to each patient before the surgical decision for four to six weeks. A shoulder MRI was performed to determine whether there was any shoulder soft tissue pathology for differential diagnosis (Table 1). The other types of shoulder pathologies, such as inflammatory rheumatic diseases and articular or bone pathologies, were excluded from the study. 

**Table 1:** Shoulder soft tissue pathologies (9)

Shoulder soft tissue pathologies
Rotator cuff tendinopathy /Impingement Syndrome
Biceps tendinopathy
Adhesive capsulitis
Calcific tendinitis
Sub-acromial bursitis
Shoulder instability
Labral tears

The patients were advised to use an orthopedic pillow before and during the conservative treatment period, as well as after the operation. Since the orthopedic pillow helps maintain cervical lordosis, it is considered a plausible choice. Furthermore, there was no significant alteration in neck pain between different types of pillows, as indicated by the post hoc analysis [11]. The orthopedic pillow is roll-shaped and contains multiple polypropylene capsules, which are pill-shaped and open-ended. The dimensions of this pillow are 50 cm in length × 45 cm in width, with a depth varying from 8 cm to 4 cm across the pillow.

In addition, images from a leading sleep position scale were shown to the patients, and information was gathered about which sleep position they most frequently assumed overnight. Since patients can change their sleeping positions during sleep, they were asked to select their most common posture. The positions were categorized based on the orientation of the shoulders relative to the pillow, not the lower extremities. These positions were divided into four groups: prone, supine, side-lying on the pain side, and side-lying on the pain-free side.

After medical treatment, the patients' visual analog scale (VAS) values were recorded both before surgery and during the first week following cervical disc herniation surgery.

Visual analog scale

VAS is a scale used for the subjective assessment of pain, allowing the person to determine the degree of pain. According to this scale, the person evaluates the amount of pain between 0 and 10. The value 0 means the absence of pain, and 10 means severe pain [12].

Statistical analysis

Descriptive statistics were expressed as frequency, percentage, mean, and standard deviation. Categorical variables were analyzed by Pearson chi-square and Fischer exact tests using 2 x 2 tables. The normality of numeric variables was tested with the Shapiro-Wilk test. The mean differences between two groups with normally distributed were compared by Student’s t-test, whereas the Mann-Whitney U test was applied to compare the not normally distributed data. Preoperative and postoperative VAS values were tested with the Wilcoxon signed rank test. The comparisons of more than two groups were tested by Kruskal Wallis variance analysis or one-way ANOVA. All statistical analyses were performed using IBM SPSS Statistics for Windows, Version 21 (Released 2012; IBM Corp., Armonk, New York, United States). p<0.05 was considered statistically significant.

## Results

A total of 72 patients who presented with unilateral pain and were diagnosed with cervical disc herniation underwent single-level ACDF surgery and were included in this study. The mean age of the patients was 46.94 (±11.56), and the distribution between males and females was equal. The preoperative mean VAS values were 7.35 (±1.06) and significantly decreased after surgery to postoperative VAS values of 3.32±1.99 (p<0.001). Twenty-one patients (29.2%) had a disc at the C3-4 level, equal to the rate at the C5-6 level. Twenty-four patients (33.3%) were at the C4-5 levels. The pain was on the right side in 37 cases (51.4%). Thirty-two cases (44.4%) slept in a side-lying position on the same side as the disc. The majority of patients (62.5%) did not opt to use an orthopedic pillow. Shoulder soft tissue pathology was observed in half of the patients (Table 2).

**Table 2:** Distribution of the general characteristics of the cases P-value was obtained from Wilcoxon signed rank test. VAS: visual analog scale.

	Values	p
Female, n (%)	36 (50%)	
Male, n (%)	36 (50%)	
Age, Mean ± SD	46.94±11.56	
Preoperative VAS, Mean ± SD	7.35±1.06	<0.001
Postoperative VAS, Mean ± SD	3.32±1.99	
Level, n( %)		
C3-C4	21 (29.2%)	
C4-C5	24 (33.3%)	
C5-C6	21 (29.2%)	
C6-C7	6 (8.3%)	
Localization, n (%)		
Left	35 (48.6%)	
Right	37 (51.4%)	
Sleep position, n (%)		
Prone	15 (20.8%)	
Side-lying on the non-disc side	15 (20.8%)	
Side-lying on the side of the disc	32 (44,4%)	
Supine	10 (13.9%)	
Orthopedic pillow use, n (%)		
Yes	27 (37.5%)	
No	45 (62.5%)	
Shoulder soft tissue pathology, n (%)		
Yes	36 (50%)	
No	36 (50%)	

General clinical characteristics of patients who underwent surgery for single-level cervical disc herniation were compared in terms of sleeping positions (Table 3). The sleeping positions of individuals at different cervical disc levels varied significantly (p=0.020). Among these, five cases with a C5-6 level disc herniation slept in either prone (33.3%) or supine (70%) positions. Eight individuals (53.3%) with a C3-4 level disc herniation more frequently slept in a side-lying position on the non-disc side. On the other hand, cases sleeping in a side-lying position on the same side as the disc were more common (40.6%) among those with disc herniation at the C4-5 levels.

**Table 3:** Comparison of the patient's general characteristics in terms of sleep positions The P value was obtained from the chi-square test, and *p was obtained from the Kruskall-Wallis test or one-way ANOVA. F: female, M; male, VAS: visual analog scale.

	Sleep positions				
	Prone	Side-lying on the non-disc side	Side-lying on the side of the disc	Supine	P
Gender					
F	5 (33.3)	7 (46.7)	20 (62.5)	4 (40)	0.247
M	10 (66.7)	8 (53.3)	12 (37.5)	6 (60)	
Age (mean±SD)	48±11.06	43.27±13	47.59±11.71	48.8±9.96	0.581^*^
Preoperative VAS (mean±SD)	7.4±0.83	7.6±0.91	7.22±1.24	7.3±1.06	0.721^*^
Postoperative VAS (mean±SD)	3.47±1.6	3.87±2.1	3.22±2.07	2.6±2.12	0.465^*^
Level					
3-4	3 (20)	8 (53.3)	8 (25)	2 (20)	0.020
4-5	4 (26.7)	6 (40)	13 (40.6)	1 (10)	
5-6	5 (33.3)	1 (6.7)	8 (25)	7 (70)	
6-7	3 (20)	0 (0)	3 (9.4)	0 (0)	
Localization					
Left	10 (66.7)	5 (33.3)	13 (40.6)	7 (70)	0.111
Right	5 (33.3)	10 (66.7)	19 (59.4)	3 (30)	
Orthopedic pillow use					
Yes	1 (6.7)	5 (33.3)	16 (50)	5 (50)	0.029
No	14 (93.3)	10 (66.7)	16 (50)	5 (50)	
Shoulder soft tissue pathology					
Yes	8 (53.3)	10 (66.7)	15 (46.9)	3 (30)	0.326
No	7 (46.7)	5 (33.3)	17 (53.1)	7 (70)	

The use of orthopedic pillows in cases with different sleeping positions was also significantly different (p=0.029). The use of orthopedic pillows was higher who sleep in the side-lying position on the same side with the disc and supine position compared to other positions. No significant correlation was found between other features and sleep position (p>0.05). Although total mean preoperative VAS scores decreased after the operation, sleep positions were not significantly relevant to the differences in VAS scores.

**Figure 1:**
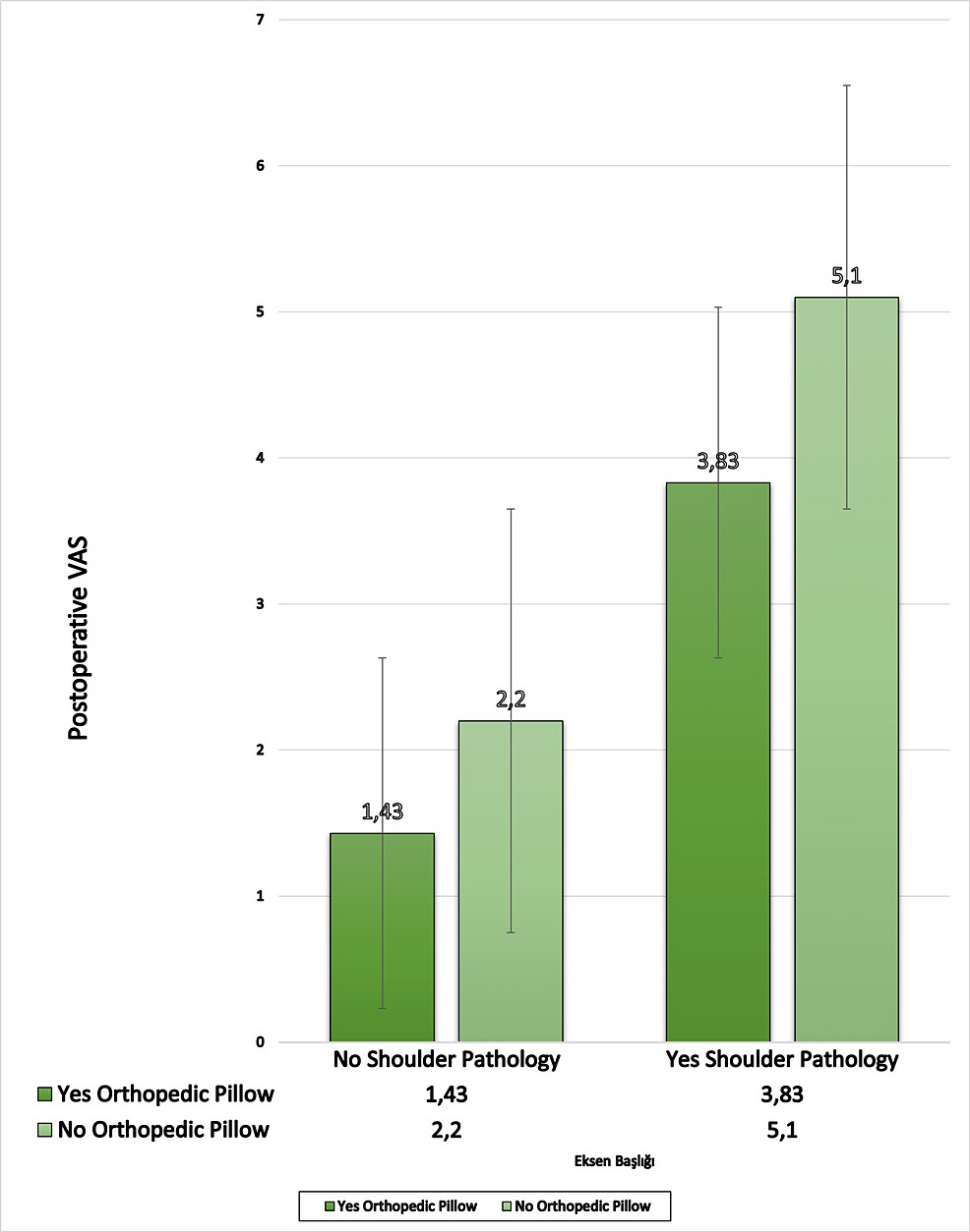
Comparison of the effect of the orthopedic pillow on postoperative VAS (visual analog scale) scores according to the presence of shoulder soft tissue pathology. It shows the postoperative VAS score is higher if there is shoulder soft tissue pathology. According to the shoulder pathology, an orthopedic pillow is slightly effective for postoperative VAS.

The general data were compared according to shoulder soft tissue pathology (Table 4). The mean preoperative VAS score was significantly higher (7.92) if there was shoulder soft tissue pathology. The mean postoperative VAS score was decreased with the use of an orthopedic pillow if there is a shoulder soft tissue pathology (p<0.05) (Figure 1).

The mean preoperative and postoperative VAS scores were significantly higher in the cases with shoulder soft tissue pathology than those without shoulder soft tissue pathology. The decrease in preoperative VAS scores to postoperative VAS scores was statistically significant (p<0.001). The study results indicate that there was no significant statistical variance observed among the levels of C3/4, C4/5, C5/6, and C6/7 concerning shoulder soft tissue pathology (p=0.139). The data revealed that the prevalence of shoulder pathology was higher at the C3/4 level in comparison to other levels. To achieve statistical significance, it may be necessary to conduct studies with larger sample sizes (Table 4).

**Table 4:** Comparison of patient's general characteristics in terms of shoulder pathology P-value was obtained from the Fisher exact test or chi-square test, and *p was obtained from the Student t-test or Mann-Whitney U test. VAS: visual analog scale.

	Shoulder soft tissue pathology		
	Yes (n: 36)	No (n: 36)	P
Preoperative VAS (mean±SD)	7.92 ± 0.87	6.78 ± 0.93	<0.001^*^
Postoperative VAS (mean±SD)	4.89 ± 1.53	1.75 ± 0.81	<0.001^*^
Level			
3-4	14 (38.9 )	7 (19.4)	0.139
4-5	13 (36.1)	11 (30.6)	
5-6	7 (19.4)	14 (38.9)	
6-7	2 (5.6)	4 (11.1)	
Orthopedic pillow use			
Yes	30 (83.3)	15 (41.7)	<0.001
No	6 (16.7)	21 (58.3)	

The preoperative and postoperative VAS scores were analyzed to compare the benefits of the orthopedic pillow in Table 5. The preoperative VAS score was significantly lower in the group that used the orthopedic pillows. Similarly, the orthopedic pillow significantly affects the postoperative VAS score (p<0.001) (Table 5).

**Table 5:** The effect of the use of an orthopedic pillow for preoperative-postoperative VAS scores *p was obtained from the Student t-test or Mann-Whitney U test. VAS: visual analog scale.

	Orthopedic pillow		
	Yes (n: 27)	No (n: 45)	P
Preoperative VAS (mean±SD)	6.74 ± 1,13	7.71 ± 0.84	<0.001^*^
Postoperative VAS (mean±SD)	1.96 ± 1.42	4.13 ± 1.84	<0.001^*^

Preoperative-postoperative VAS differences, shown in Table 6, were 4.78±1.31 in those who used orthopedic pillows and 3.58±1.42 in those who did not. They were statistically significantly higher in cases using orthopedic pillows (<0.001). In addition, the preoperative-postoperative VAS difference was statistically significantly higher in individuals without shoulder soft tissue pathology. The preoperative-postoperative VAS differences were similar regarding sleep positions (p>0.05) (Table 6).

**Table 6:** Examination of the change of preoperative VAS scores to postoperative VAS scores The P value was obtained from the Kruskal-Wallis test or one-way ANOVA. p was obtained from the Student t-test or Mann-Whitney U test. VAS: visual analog scale.

	The Change in Preoperative and Postoperative VAS Scores	
	Mean±SD	p
Sleep positions		
Prone	3.93±1.16	0,450
Side-lying on the non-disc side	3.73±1.62	
Side-lying on the side of the disc	4±1.55	
Supine	4.7±1.57	
Orthopedic pillow use		
Yes	4.78±1.31	<0,001
No	3.58±1.42	
Shoulder soft tissue pathology		
Yes	3.03±1.18	<0,001
No	5.03±1.03	

## Discussion

Cervical disc herniation is a common cervical spine disease that can cause arm or shoulder pain. The calculated annual incidence can vary but is approximately 18 per 100,000 [8]. Pain can be caused by cervical radiculopathy. The yearly prevalence was estimated to be 83/100,000, peaking in the fourth and fifth decades according to a large epidemiological study applying comprehensive criteria, which included neck and arm pain and corresponding MRI findings indicating that one or more nerve roots were affected [13]. Pain can initially start in the neck or shoulder and spread through the fingers along the arm. Cervical disc herniation may lead to pain depending on the level of the cervical disc involved. The diagnosis of cervical disc herniation is typically made using magnetic resonance imaging (MRI).

Most patients with cervical radiculopathy can be treated nonsurgically [14]. A recent randomized controlled trial comparing conservative treatments showed that improvement at 6 and 12 weeks was significantly better in those who received physiotherapy or wore a collar [15]. Twenty-six percent of the patients underwent surgery, and the well-established indication for surgery was a combination of radicular pain, sensory loss, and muscle weakness [16].

The most frequent level of cervical disc herniation is reported as C6-7 (69%), followed by C5-6 (19%) in the literature. The least frequent level is C4-5, reported at 2% [17]. In the present study, C4-5 disc herniation was the most common, at a rate of 33.3%, likely because we reviewed patients with shoulder/arm pain. C6-7 disc herniations accounted for 8.3% since pain from this level rarely affects the shoulder/arm. Additionally, non-operated patients were excluded from the study, and only those who underwent surgery for cervical disc herniation were included.

C4-5 level discs can cause C5 root symptoms and usually present with shoulder/arm pain. The exact point of the pain may not be estimated because it may radiate from the neck to the arm. It may manifest with tenderness and pain along this tracing [18]. That is why patients cannot differentiate the localization of the pain exactly. 

Shoulder/arm pain can be associated with cervical disc herniation and shoulder pathologies; therefore, a differential diagnosis is necessary. Self-reported prevalence of shoulder pain is estimated to be between 16% and 26% [19]. Physical factors such as lifting heavy loads, repetitive movements in uncomfortable positions, and vibrations influence symptoms and disability [20]. 

Shoulder soft tissue pathologies such as rotator cuff tendon impingement, biceps tendinopathy, adhesive capsulitis, calcific tendinitis, sub-acromial bursitis, and labral tears must be kept in mind for distinction [21]. Likewise, painful impingement syndromes of the shoulder joint are detected in approximately 24% of patients with cervical radiculopathy [22]. Since pain is a subjective assessment, imaging methods can distinguish these two pathologies. While a cervical MRI shows cervical discographies, an MRI of the shoulder joint will help us understand the presence of shoulder soft tissue pathologies. Thus, it can help to make a differential diagnosis in patients with cervical disc herniation when making an operation decision or to evaluate post-operative pain. This study detected the coexistence of shoulder soft tissue pathology and cervical disc herniation as 50%. Total mean VAS scores were significantly reduced after cervical disc herniation operations, as they should be even if shoulder pathologies were present. In this case, it has been observed that cervical disc herniation operations effectively reduce pain. Therefore, if the operation indications are encountered in cervical disc herniation, it should be operated on regardless of shoulder soft tissue pathology.

Axial loading is an etiological factor for cervical disc herniation, as seen in workers carrying heavy weights. Presumably, biomechanics of the upper extremity may cause this dual pathology. Likewise, if the cervical muscles are not strong enough, it will increase the load on the discs and cause herniation [13]. On the other hand, the effects of neck and spine positions and shoulder strain have also been studied during sleep, which covers approximately one-third of the population's lifespan [23]. Previous studies have shown that sleeping positions may cause shoulder pathologies. Pressure on the shoulder and insufficient blood flow can be counted among these reasons [24]. The most common sleep positions were investigated, and the eventuation of shoulder soft tissue pathologies was evaluated. In a study on shoulder pathologies, the supine position was reported as advantageous, while the other three positions were harmful to the shoulder due to high pressure [23]. In this present study, we divided the sleeping positions as prone, spine, and side-lying. In order to examine the effect of side-lying position on cervical disc herniation, we evaluated it by separating it as lying position on the disc or the opposite side of the disc. The side-lying position is significantly high in cervical disc hernias and shoulder pathologies. From this result, lying on the side can be considered a predisposing factor for both shoulder soft tissue diseases and neck hernia.

Our study determined that there were different levels of cervical disc herniation in different sleeping positions. The patients tend to lie on the contrary side of the disc at the C3-4 level. They lie on the same side of the disc at the C4-5 level. Whether the lying position causes cervical disc herniation or whether the sleeping positions of the patients change due to pain is an unknown situation. In this regard, studies on long-term sleep position changes can be conducted in the future. There was no difference between the sleep positions regarding preoperative and postoperative VAS scores. Sleeping positions do not affect the degree of shoulder/arm pain before the operation, and they do not affect the pain after the operation.

On the other hand, the presence of shoulder soft tissue pathologies significantly affects pre- and postoperative pain. The pain did not decrease sufficiently in shoulder soft tissue pathology patients after cervical disc herniation operation. Therefore, the specialist should remember that if the postoperative pain after cervical disc surgeries does not decrease as desired, there is a high probability of accompanying shoulder soft tissue pathology.

Improving sleep quality is effective in reducing pain according to spinal problems [25,26]. In addition, the effectiveness of pillow types is being studied to increase sleep quality and indirectly reduce pain [11]. The use of appropriate pillows may be effective in reducing neck pain, which can change the heart rate and also deep and quality sleep. Our study examined the effect of orthopedic pillow use on preoperative and postoperative pain. It is also recommended to do a separate study on memory foam or feather pillows, which are known to affect pain. 

In this study, it is understood that using orthopedic pillows does not contribute to the formation of cervical disc herniation and, therefore, the use of them is not necessary to prevent cervical disc herniation. Although an orthopedic pillow may not prevent cervical disc herniation, preoperative pain was significantly less in those using orthopedic pillows. At the same time, the postoperative pain was less in those who used orthopedic pillows. The use of orthopedic pillows has a positive effect on the difference in pain before and after the operation, while the presence of shoulder pathology has a negative effect. It is slightly effective in improving postoperative VAS scores in cases with shoulder pathology, although the difference is not statistically significant.

Limitations

In our study, personal questions about routine daily life were asked. These results were obtained by asking questions to the patients during the control. One-to-one follow-up is required for more reliable follow-up.

## Conclusions

The presence of shoulder pathologies can be confused with symptoms of cervical disc herniation and can also impact postoperative pain after cervical disc surgery. Shoulder soft tissue pathologies should be taken into account when postoperative shoulder pain doesn't decrease as expected following cervical disc surgeries. The use of orthopedic pillows is effective for both preoperative and postoperative pain but does not contribute to the causation or aggravation of shoulder pathologies or cervical disc herniations. Orthopedic pillows should be recommended to patients both before and after surgery. This study found no significant correlation between sleep position and the intensity of preoperative pain or postoperative pain levels. The influence of sleeping positions on managing patients' pain is negligible. Different sleeping postures may lead to disc herniations at distinct levels.
